# In-depth exploration of the shared genetic signature and molecular mechanisms between end-stage renal disease and osteoporosis

**DOI:** 10.3389/fgene.2023.1159868

**Published:** 2023-11-24

**Authors:** Weijuan Lou, Wenhui Li, Ming Yang, Chong Yuan, Rui Jing, Shunjie Chen, Cheng Fang

**Affiliations:** ^1^ Shanghai Sixth People’s Hospital Affiliated to Shanghai Jiao Tong University School of Medicine, Shanghai, China; ^2^ Department of Gynecology and Obstetrics, Changhai Hospital, Second Military Medical University, Shanghai, China; ^3^ Department of Nephrology, Shanghai Fourth People’s Hospital, Shanghai, China; ^4^ Department of Pathology, School of Basic Medicine, Fudan University, Shanghai, China; ^5^ Department of Nephrology, Xijing Hospital, The Fourth Military Medical University, Xi’an, China; ^6^ Department of Hepatic Surgery IV, Eastern Hepatobiliary Surgery Hospital, Second Military Medical University, Shanghai, China

**Keywords:** diagnostic gene, end stage renal disease, osteoporosis, machine learning, weighted gene co-expression network analysis

## Abstract

**Background:** Osteoporosis (OS) and fractures are common in patients with end-stage renal disease (ESRD) and maintenance dialysis patients. However, diagnosing osteoporosis in this population is challenging. The aim of this research is to explore the common genetic profile and potential molecular mechanisms of ESRD and OS.

**Methods and results:** Download microarray data for ESRD and OS from the Gene Expression Omnibus (GEO) database. Weighted correlation network analysis (WGCNA) was used to identify co-expression modules associated with ESRD and OS. Random Forest (RF) and Lasso Regression were performed to identify candidate genes, and consensus clustering for hierarchical analysis. In addition, miRNAs shared in ESRD and OS were identified by differential analysis and their target genes were predicted by Tragetscan. Finally, we constructed a common miRNAs-mRNAs network with candidate genes and shared miRNAs. By WGCNA, two important modules of ESRD and one important module of OS were identified, and the functions of three major clusters were identified, including ribosome, RAS pathway, and MAPK pathway. Eight gene signatures obtained by using RF and Lasso machine learning methods with area under curve (AUC) values greater than 0.7 in ESRD and in OS confirmed their diagnostic performance. Consensus clustering successfully stratified ESRD patients, and C1 patients with more severe ESRD phenotype and OS phenotype were defined as “OS-prone group”.

**Conclusion:** Our work identifies biological processes and underlying mechanisms shared by ESRD and OS, and identifies new candidate genes that can be used as biomarkers or potential therapeutic targets, revealing molecular alterations in susceptibility to OS in ESRD patients.

## Introduction

Chronic kidney disease (CKD) is commonly associated with mineral and bone disorders, osteoporosis, and an increased risk of fractures (Wu et al., 2022). Eighty-five percent of women with osteoporosis have mild to moderate renal impairment, and because of the strong correlation between osteoporosis and CKD (Klawansky et al., 2003), it is important to treat osteoporotic patients effectively and safely with renal insufficiency without any adverse effects on intrinsic renal function (Broadwell et al., 2021).

Bone disease in ESRD is a mixture of decreased bone density and impaired bone quality due to microtrauma as well as microarchitectural and collagen disturbances. It is associated not only with an increased risk of fracture, but also with poor nutritional status with decreased muscle strength and low lean body mass, and increased vascular calcification (Hobson et al., 2019), yet many basic research questions remain unanswered. Less bone mineralization is associated with an increased risk of vascular heterotopic calcification and its clinical sequelae, known as the “calcification paradox” (Persy and Haese, 2009). Vascular calcification with vascular “ossification” may be the result of impaired bone remodeling and osteogenesis driven by osteogenic transcription factors, such as runt-related transcription factor 2 (RUNX2) and Msh homeobox 2 (Bucay et al., 1998; Shao et al., 2005; Sun et al., 2012). Similarly, *in vitro* studies have shown that vascular smooth muscle cells (VSMC) and perivascular cells can undergo osteogenic differentiation and produce osteogenic transcription factors and proteins in response to high concentrations of phosphate, calcium, glucose, oxidized lipids, Inflammatory cytokines and various toxins (Shanahan, 2013). Therefore, a comprehensive review of potential targets based on the common pathogenesis between ESRD and OS may benefit the development of future treatments.

With the rapid development of gene chip technology, researchers can quickly measure the expression of thousands of gene data in various diseases, which will help people gain a deeper understanding of the pathogenesis of diseases from the genetic level. Common transcriptional signatures may provide new insights into the common pathogenesis of ESRD and OS. The purpose of this study was to identify the central genes associated with the pathogenesis of ESRD complicated with OS, and to try to determine their diagnostic ability for OS in ESRD patients.

## Methods and materials

### Data collection and processing

End-stage renal disease as well as osteoporosis datasets were obtained through the Gene Expression Omnibus (GEO) database. Screening was performed by the following criteria: i) the gene expression profiles must include cases and controls; ii) the tissues used for sequencing were all derived from the same tissue; iii) the number of samples per group should not be less than 20 to ensure the accuracy of WGCNA. Finally, GSE97709, GSE37171 were included in the study as ESRD dataset and GSE56814, GSE56815 as OS dataset. Details of the dataset are provided in Supplementary Table S1.

### Weighted gene co-expression network analysis

Weighted gene co-expression network analysis (WGCNA) (Langfelder and Horvath, 2008) is an algorithm that can discover co-expressed gene modules with high biological significance and explore the relationship between gene networks and diseases. More than 20,000 genes were sequenced in the GEO dataset, and most of these genes had no expression differences between samples, so we selected the top 25% of genes with large variance based on variance for WGCNA analysis to obtain ESRD and OS-related modules. First, all samples are clustered based on gene co-expression similarity and outliers are removed. Second, the best soft threshold is selected using the power function pickSoftThreshold. In addition, modules with a cut height of 0.25 are merged with a minimum module size of 100 genes. The expression profile of each module was summarized by module signature genes (ME) and correlations between ME and clinical features were calculated.

### Machine learning to screen candidate genes

We performed RF analysis and LASSO regression using the R packages “random forest” and “glmnet”. Two machine learning algorithms, RF (Newman et al., 2019) and LASSO (Liberzon et al., 2015), were used to further filter candidate genes for ESRD and OS diagnosis. LASSO is a regression method that has shown superiority in evaluating high-dimensional data. We used the RF algorithm to initially screen diagnostic genes with importance scores greater than 0. Among the obtained genes, the LASSO algorithm was used to further reduce the dimensionality to obtain the final diagnostic genes.

### Patient sample collection and molecular validation

To validate our identification of the diagnostic role of the 8hub gene in ESRD and OS. We analyzed human serum samples collected from patients with ESRD (n = 17) or OS (n = 21) and patients with ESRD with OS (n = 15) from the Sixth People’s Hospital of Shanghai. The protocol for human samples was approved by the Clinical Ethics Committee of the Sixth People’s Hospital of Shanghai Jiao Tong University. Serum samples were obtained from patients with ESRD with OS and from patients with ESRD without OS. In addition, serum CPNE7 and MFGE8 levels were measured using the indicated ELISA kits (mlbio, Shanghai, China) according to the manufacturer’s protocol.

### Immuno-infiltration analysis

We measured the relative abundance of each cellular infiltrate in the tumor microenvironment (TME) using the single sample genomic enrichment analysis (ssGSEA) technique (Barbie et al., 2009). The genomes identifying the 23 immune cells infiltrating the TME were collected from an earlier study (Zhang et al., 2020). The “corrplot” package in the R language mapped the correlation heat map revealing the correlation between core genes and infiltrating immune cells.

### Common miRNAs-target gene network construction

TargetScan predicts miRNA biological targets by searching for the presence of conserved 8mer, 7mer and 6mer loci matching each miRNA seed region (McGeary et al., 2019). The intersection of shared miRNAs in ESRD and OS with miRNAs corresponding to core genes was used to construct miRNA-mRNA regulatory networks. cytoscape (Shannon et al., 2003) software was used to visualize the networks.

### Statistical analysis

All statistical tests were performed using R software version 4.1.2. Wilcoxon was used to analyze the differences between the two groups. Correlations between variables were determined using Pearson or Spearman correlation tests. Statistical significance was set at two-tailed *p* < 0.05.

## Results

### Identifying shared transcriptomic signatures between ESRD and OS

**FIGURE 1:**
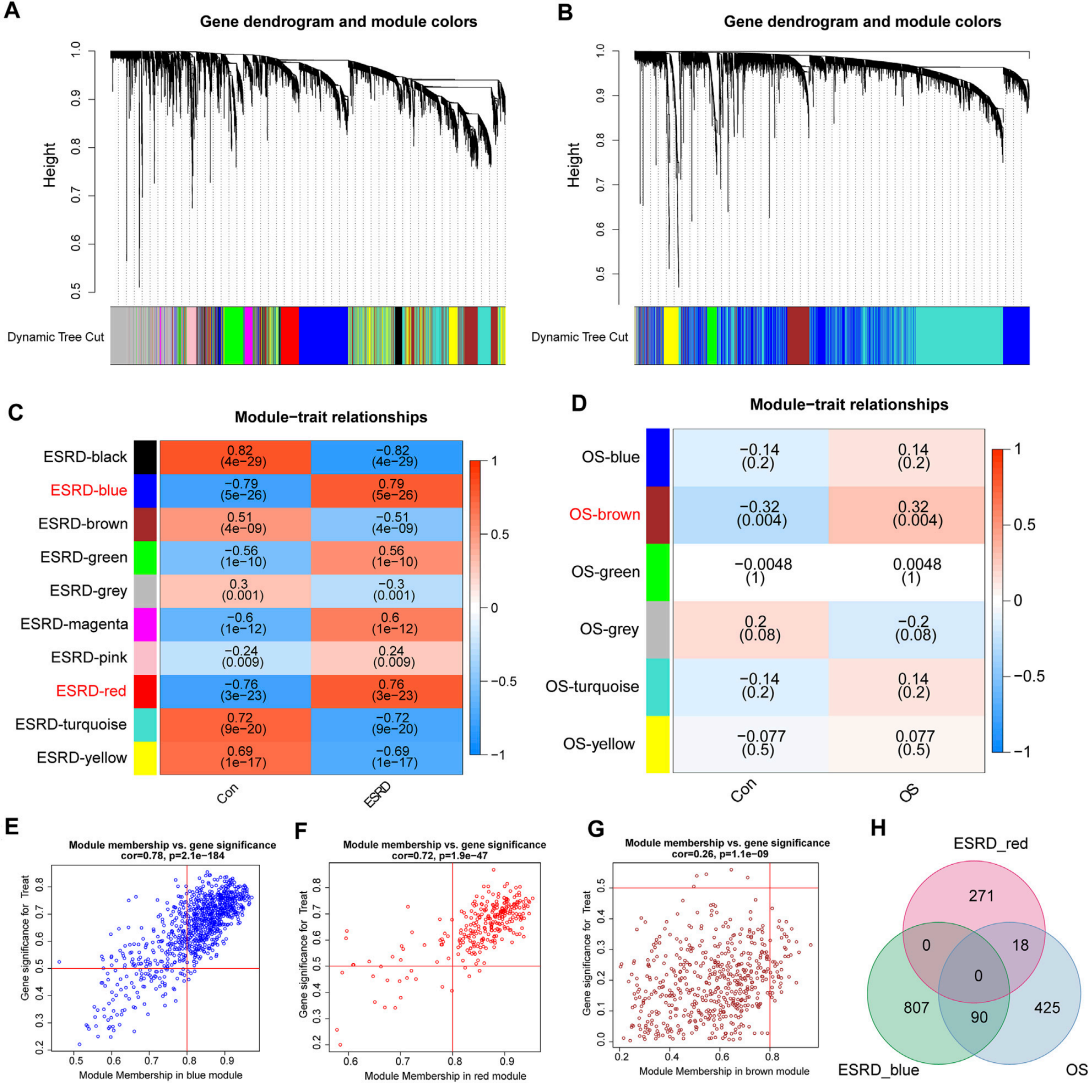
Weighted Gene Co-expression Network Analysis (WGCNA). **(A)** Cluster dendrogram of ESRD co-expressed genes. **(B)** Cluster dendrogram of OS co-expressed genes. **(C)** Module-feature relationships in ESRD. **(D)** Module-trait relationships in OS, with each cell containing the corresponding correlation and *p*-value. Characteristic plots of **(E)** blue module vs **(F)** red module GS vs MM values in ESRD. **(G)** Characteristic plot of GS vs MM values of the brown module in OS. **(H)** Overlapping shared genes between the blue and red modules of ESRD and the brown module of OS.

According to the previously set criteria, the discovery cohorts numbered GSE37171, GSE56815 were selected as shared genes. A total of 11 modules were identified in GSE37171 by WGCNA, and each color represents a different module (Figure 1A). Then, draw a heat map on the module-trait relationship according to the Spearman correlation coefficient to evaluate the association between each module and the disease. The “blue” and “red” modules are highly positively associated with ESRD, including 898 and 290 genes, were selected as ESRD-related modules (blue modules: R = 0.79, P = 5e-26; red modules: R = 0.76, P = 3e-23) (Figure 1C). Likewise, a total of 6 modules were identified in GSE56815 (Figure 1B), and the module “brown” was the only one positively correlated with OS (R = 0.32, *p* = 0.004), including 534 genes (Figure 1D). Further, the above three modules were used as key modules for gene significance (GS) and module membership (MM) analysis, and the correlation coefficients between GS and MM of the blue, red and brown modules were all significantly positively correlated (R > 0.2; *p* < 0.001) (Figures 1E–G). The positive correlation module for ESRD and OS had 108 overlapping genes, defined as “shared genes” (Figure 1H) and highly relevant to the pathogenesis of ESRD and OS.

### Analysis of functional features associated with common pathogenesis

**FIGURE 2:**
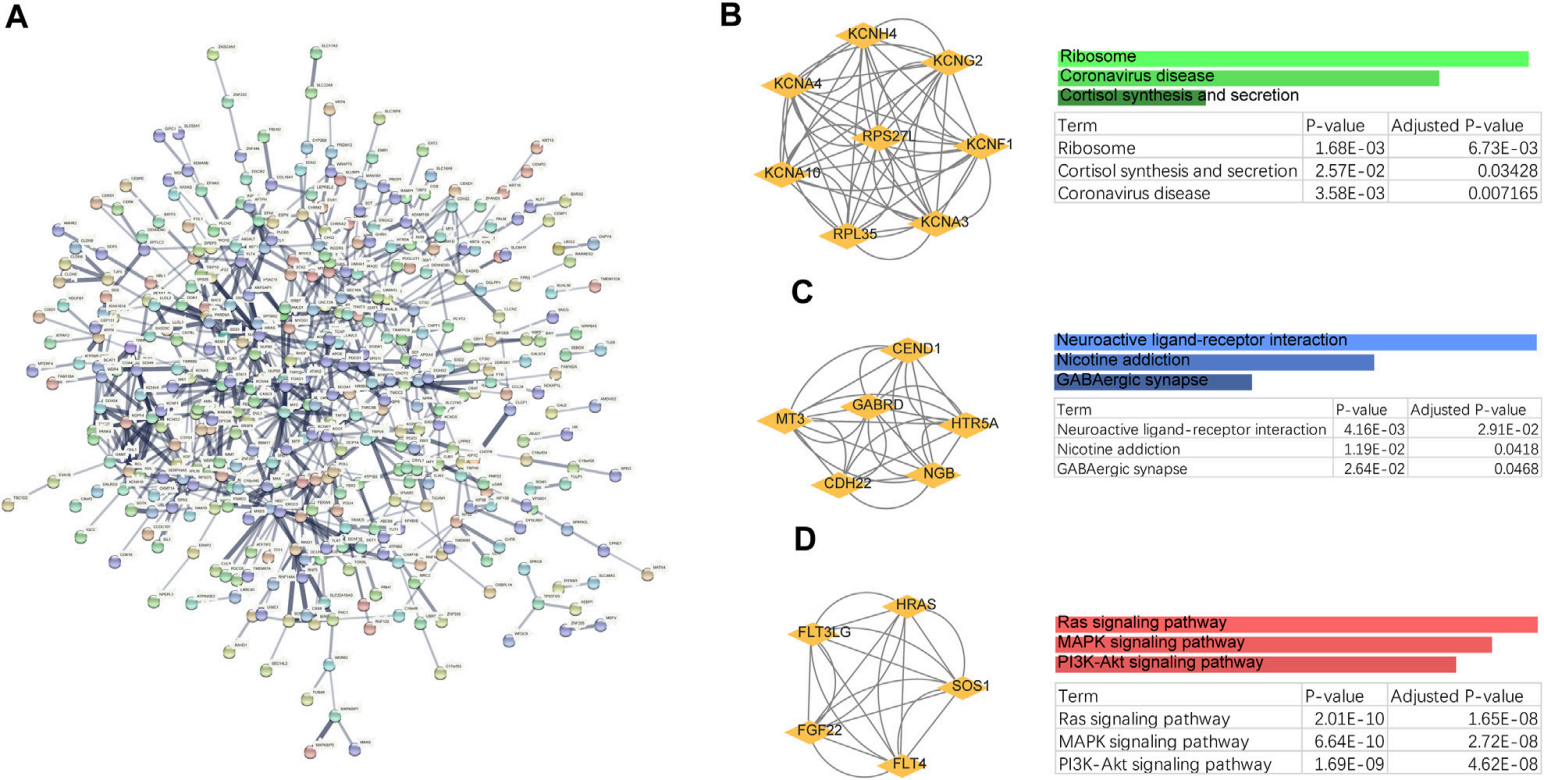
PPI networks and biological functions of shared genes. **(A)** PPI networks of shared genes. **(B–D)** Functional enrichment analysis of three major clusters identified by MCODE analysis.

To explore the potential functions of the shared genes, we further constructed a protein-level PPI network (Figure 2). Three main clusters were extracted using MCODE analysis. For each gene cluster, three keywords were selected to summarize its main biological functions. Functional enrichment analysis showed that genes in cluster 1 were mainly related to Ribosome, Cortisol synthesis and secretion, and genes in cluster 2 were closely related to Neuroactive ligand-receptor interaction. Cluster 3 mainly involves Ras signaling pathway, MAPK signaling pathway, PI3K-Akt signaling pathway. Impairment of Ras/MAPK/ERK signaling has been shown to promote ESRD and OS (Sharma et al., 2013; Donate-Correa et al., 2021). Thus, these results strongly suggest a common biological process in the development of these two diseases.

### Identifying candidate hub genes in ESRD and OS using machine learning

**FIGURE 3:**
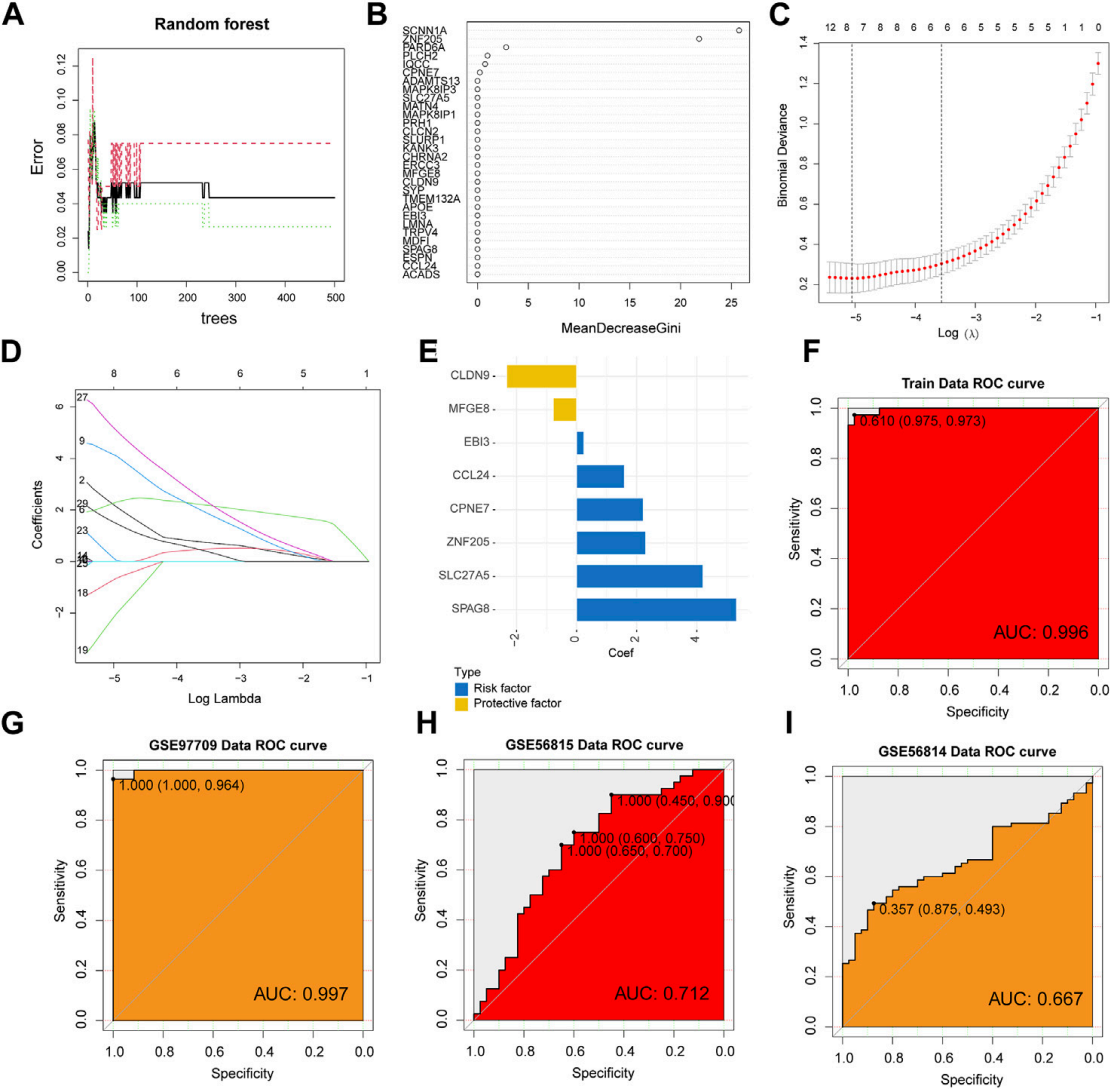
Machine Learning Screening Genes and Modeling. **(A)** Random forest error rate plot for candidate genes screened primarily. **(B)** Variable importance score plot. **(C)** Cross-validation for selecting the best tuning parameter log(Lambda) in LASSO regression analysis. **(D)** LASSO coefficient profiles of candidate genes. **(E)** The coefficient of each core gene in the model. ROC curve analysis of ESRD training set **(F)** and test set **(G)**. **(H, I)** ROC curve analysis in the OS cohort.

To further screen the core genes, we used the random forest method to initially screen 30 candidate genes from 108 genes (Figures 3A,B), and then further dimensionality reduction by LASSO to obtain the final 8 core shared genes (Figures 3C,D), namely, ZNF205, CPNE7, SLC27A5, MFGE8, CLDN9, EBI3, SPAG8, CCL24. Based on the coefficients of these genes, we calculated an 8-gene signature score for each patient (Figure 3E). The area under the ROC curve of the gene features in the ESRD training set was 0.996, and also in the validation set (GSE97709), the area under the ROC curve was 0.997, confirming the diagnostic value in ESRD diseases (Figures 3F,G). We also verified the diagnostic effect of the 8-gene signature in the OS cohort. The ROC of the training set and the verification set were 0.712 and 667 (Figures 3H,I), respectively, indicating that the model is also applicable to OS.

### Immune infiltration analysis in patients with ESRD and OS

**FIGURE 4:**
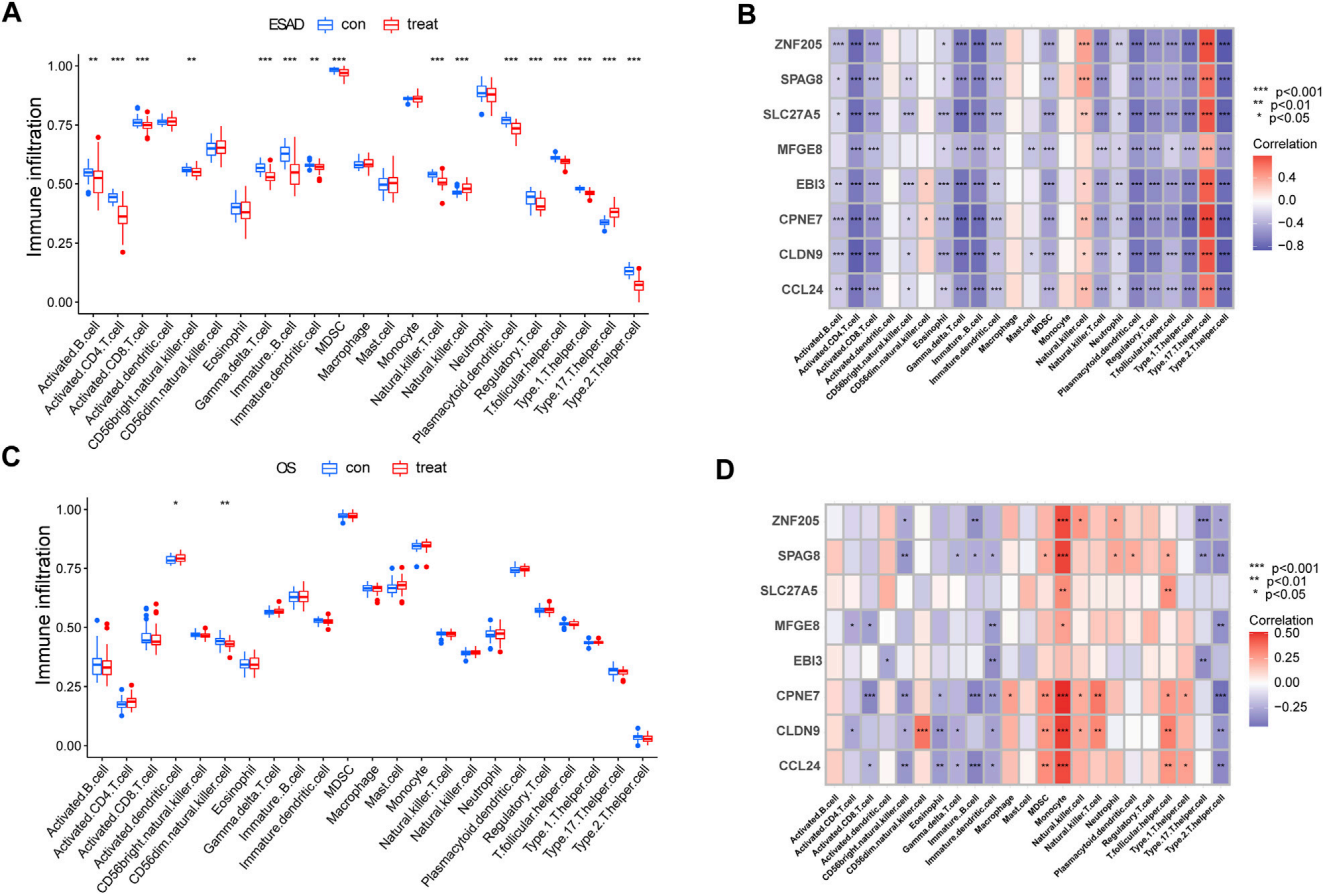
Analysis of Immune Cell Infiltration in ESRD and OS Patients. **(A)** Differences in immune infiltrating cells in patients with ESRD. **(B)** Immune infiltration analysis of 8 candidate genes in ESRD. **(C)** Differences in immune infiltrating cells in OS patients. **(D)** Immune infiltration analysis of 8 candidate genes in OS.

As persistent inflammation and immune dysfunction are very common features of chronic kidney disease (CKD) leading to ESRD (Liu et al., 2022), while osteoporosis, although a bone metabolic disease, may be mediated by chronic inflammation (Suzuki, 2019). Therefore, we sought to analyze the correlation of 8 hub genes in immune cells in ESRD *versus* OS. ssGSEA calculated the relative content of immune cells in ESRD *versus* OS. patients with ESRD exhibited a lower proportion of CD4^+^ T cells as well as CD8^+^ T cells, and a higher proportion of Th17 cells (Figure 4A). Patients with OS exhibited a higher proportion of dendritic cells, and a lower proportion of CD56dim NK cells (Figure 4C). The results of the correlation analysis showed that the 8 core genes may have opposite or nonsignificant effects on some immune cells in ESRD and OS. For example, in ESRD, the 8 genes had a broad negative correlation with CD4^+^ T cells as well as Th2 cells, and a broad positive correlation with Th17 cells (Figure 4B). In contrast, some hub genes in OS were negatively correlated with both Th17 and Th2 cells, while broadly positively correlated with monocytes (Figure 4D). Although we did not find immune features common to ESRD and OS, these 8 core genes have similar or opposite immune functions in ESRD and OS.

## Gene signatures can stratify ESRD patients

**FIGURE 5:**
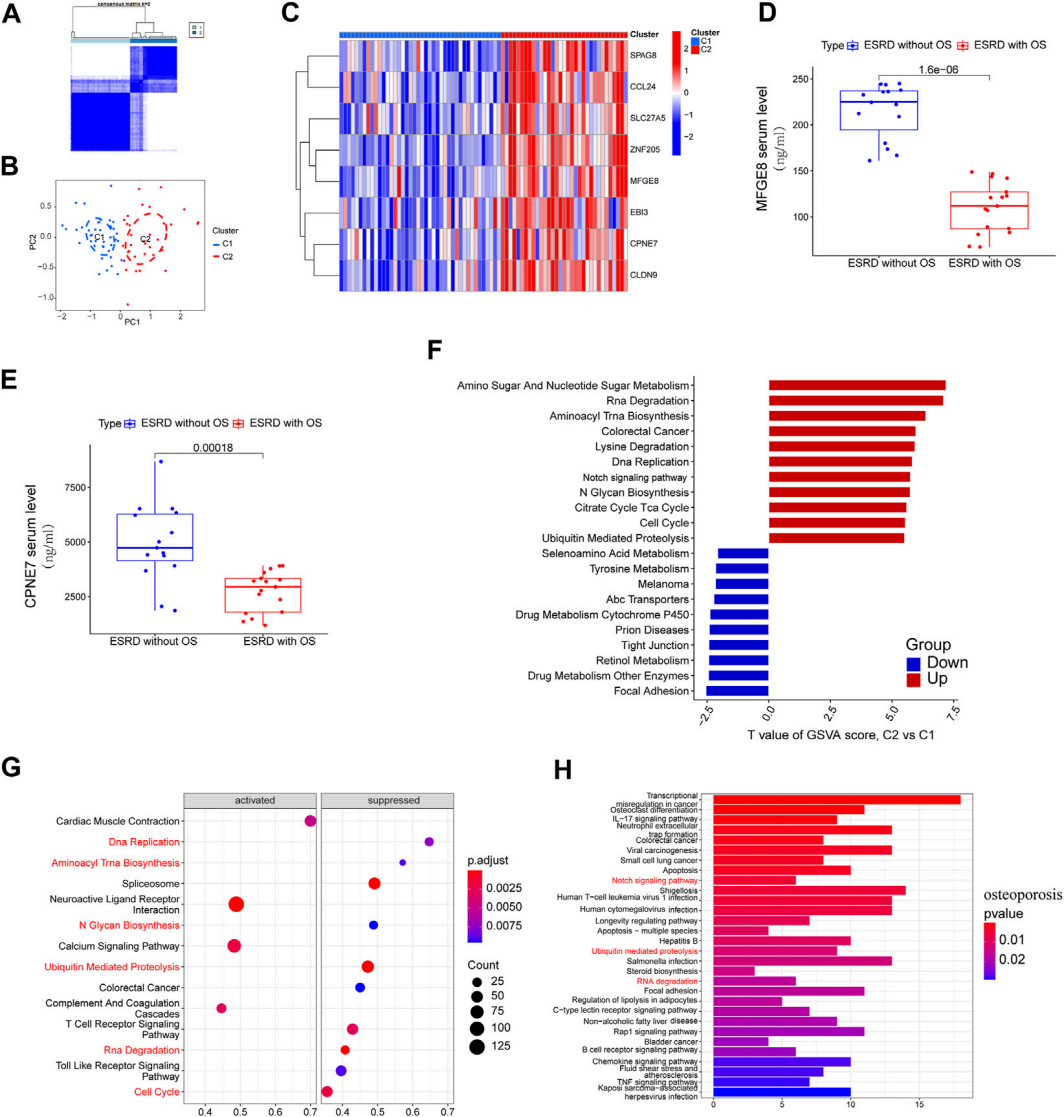
Stratified analysis of OS susceptibility in patients with ESRD. **(A)** Consensus clustering divides ESRD patients into C1 and C2 clusters. **(B)** PCA analysis of C1 and C2 clusters. **(C)** Expression of 8 hub genes in clusters C1 and C2 clusters. ELISA analysis showed elevated serum **(D)** MFGE8 and **(E)** CPEN7 levels in ESRD patients without OS *versus* ESRD patients with OS. **(F)** GSVA analysis of C1 and C2 clusters. **(G)** GSEA analysis of ESRD patients, pathways are divided into activation and inhibition for display. **(H)** KEGG enrichment of downregulated genes in OS patients.

Due to the strong diagnostic power and similar biological processes and immune signatures of the 8-gene signature in ESRD and OS diseases. We therefore sought to determine whether an 8-gene signature could identify ESRD patients predisposed to OS. Using a consensus clustering algorithm, we divided ESRD patients into clusters C1 and C2 (Figure 5A). PCA analysis confirmed that the C1 and C2 clusters had distinct two-dimensional distribution patterns (Figure 5B). Eight genes had significantly high expression in the C2 cluster compared with the C1 cluster (Figure 5C), which may indicate that the C1, C2 clusters have completely different biological phenotypes. In addition, MFGE8 and CPEN7 were detected in serum by ELISA and were significantly lower in patients with ESRD with OS compared to those with ESRD alone (*p* < 0.05) (Figures 5D,E). The GSVA algorithm shows the most significantly altered KEGG terms for the C1, C2 cluster, with the C2 cluster having higher Amino Sugar And Nucleotide Sugar Metabolism, Rna Degradation, Aminoacyl Trna Biosynthesis and lower Focal Adhesion, Drug Metabolism (Figure 5D). We performed GSEA analysis (DEGs is provided in Supplementary Table S2) in ESRD patients and found that a large number of KEGG terms were enriched in ESRD, such as Rna Degradation, Aminoacyl Trna Biosynthesis were inhibited in ESRD (Figure 5E), suggesting that the C1 cluster had a more severe ESRD phenotype, while the C2 group had a lesser degree of ESRD. We performed KEGG analysis of differential OS downregulated genes (Supplementary Table S3) and found significant enrichment of Ubiquitin mediated proteolysis, Notch signaling pathway and RNA degradation (Figure 5F). These results suggest that the C1 cluster has a more pronounced OS profile and is considered as an “OS-prone group”, while the C2 cluster is considered as a “not-prone OS group".

### Identification and network construction of shared miRNAs in ESRD and OS

**FIGURE 6:**
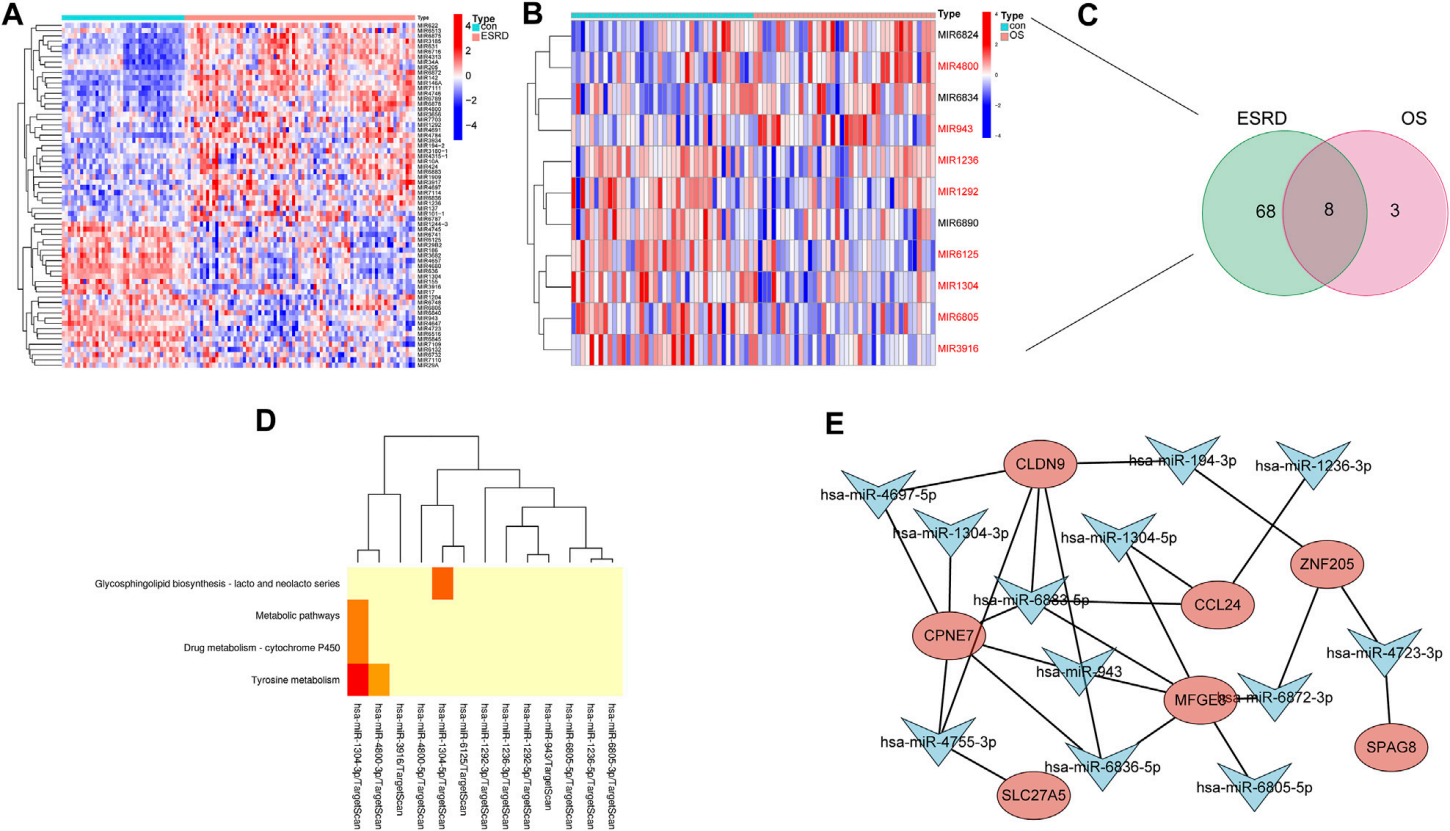
Construction of shared miRNA-mRNA network in patients with ESRD and OS. Heatmap of differential miRNA profiling in **(A)** ESRD and **(B)** OS patients. **(C)** miRNAs shared between ESRD and OS patients. **(D)** Enrichment analysis of shared miRNAs. **(E)** Shared miRNA-mRNA network construction.

We identified 66 and 11 miRNA precursors (Figures 6A,B) in GSE37171 (ESRD cohort) and GSE56815 (OS cohort), respectively, based on the screening criteria. Eight of the miRNA precursors were considered as “shared miRNAs” (Figure 6C). The miEAA database was used for maturation body conversion and enrichment analysis of these eight miRNA precursors, which were found to be mainly associated with various metabolic pathways, including complexine metabolism, drug metabolism-cytochrome P450 and other metabolic pathways, consistent with the C1 cluster profile (Figure 6D). These results further strengthen the common disease features of ESRD and OS.

Since these 8 hub genes are involved in common biological processes with differential miRNAs for ESRD and OS (8 miRNA precursors), we sought to construct miRNA-mRNA regulatory networks to elucidate the disease processes common to ESRD and OS. The 1,049 potential miRNA targets of 8 hub genes were predicted by TargetScan. Among them, 12 matrices with 8 miRNA precursors (hsa-miR-194-3p, hsa-miR-6805-5p, hsa-miR-6883-5p, hsa-miR-6872-3p, hsa-miR-943, hsa-miR-1236-3p, hsa-miR-1304-3p hsa-miR-1304-5p, hsa-miR-6836-5p, hsa-miR-4755-3p, hsa-miR-4723-3p, hsa-miR-4697-5p) existed intersections. Finally, the miRNAs-mRNAs network was constructed, including 19 nodes (12 miRNAs, 7 mRNAs) and 25 edges (Figure 6E).

## Discussion

The kidney plays an important role in the systemic regulation of mineral metabolism, and the occurrence of secondary OS in patients with CKD or ESRD has been clinically documented for a long time (Kooman et al., 2014). According to a meta-analysis of prospective cohort studies, low bone mineral density (BMD) levels in ESRD patients are associated with increased cardiovascular disease-related mortality and all-cause mortality (Qu et al., 2013). In patients with ESRD, this is due to alterations in the osteo-vascular axis and metabolic and hormonal abnormalities associated with CKD-MBD, such as disorders of mineral metabolism, vitamin D deficiency, secondary hyperparathyroidism and excess or molecular defects affecting bone formation (Vervloet et al., 2014; Evenepoel et al., 2015). However, few studies seem to have explored susceptibility aspects of OS in ESRD at the genetic level. We explored for the first time the common mechanisms of ESRD and OS using WGCNA and identified ESRD patients susceptible to OS based on consensus clustering.

### Common transcriptomic features and functional characteristics associated with ESRD and OS pathogenesis

In order to explore the common effector genes of both diseases, we obtained the intersection of co-expression modules using WGCNA and identified three major signals, RAS, MAPK, and PI3K/AKT, by PPI network and gene cluster identification, and found that gene cluster 3 is involved in three major signals, RAS, MAPK, and PI3K/AKT. pathway, Ganoderic acid prevents renal fibrosis by inhibiting the MAPK signaling pathway. Meanwhile, several studies have confirmed that silencing the MAPK/NF-κB signaling pathway leads to reduced osteoclastogenesis, thereby attenuating OS (Xiao et al., 2020; Yang et al., 2022). These studies are consistent with our findings. It also seems unsurprising that ribosomes are highly enriched in gene cluster 1, and that bitter ginseng derivatives can inhibit osteoclastogenesis through ribosomal protein s5 (RPS5) and by regulating PI3K/Akt, NF-κB and MAPK pathways in osteoclastogenesis (Chen et al., 2017; Xin et al., 2018). In addition, targeting the RPL31-mediated RAS/MEK/ERK signaling pathway inhibited osteogenic differentiation of human bone marrow mesenchymal stem cells (Peng et al., 2022). Although, the relationship between renal fibrosis and ribosomal proteins has not yet been elucidated, our results provide theoretical support for the involvement of ribosomal proteins in regulating CKD progression.

### Hub 8 gene as a diagnostic signature for OS susceptibility in ESRD patients

The management of osteoporosis in patients with advanced CKD or dialysis fundamentally revolves around diagnosing and targeting mineralization and renewal abnormalities with the goal of improving bone density, volume and quality. However, for the detection of OS occurrence, only limited tools are available, such as BMD tests, bone turnover markers (BTM) (Ginsberg and Ix, 2022). Both high and low bone turnover lead to low bone mass and fracture risk, but the treatment strategies are diametrically opposed (Iimori et al., 2012). The most commonly used BTM in the clinical setting is intact parathyroid hormone (iPTH). Although a target level of iPTH of 150 pg/mL or 2-9 times the local laboratory reference range has been suggested, the most recent KDIGO guidelines do not recommend a specific iPTH because iPTH concentrations do not consistently reflect bone structure and bone turnover histology (Isakova et al., 2017). Our work identifies a potential approach that can be used as a potential indicator of OS susceptibility in ESRD patients. First, we obtained 8 shared genes (ZNF205, CPNE7, SLC27A5, MFGE8, CLDN9, EBI3, SPAG8, CCL24) by a machine learning approach. ROC analysis showed that they have good predictive effect in ESRD and OS. Crucially, consensus clustering based on 8hub genes successfully classified ESRD patients into C1 (OS-prone group) and C2 (not-prone OS group). Abc transporters as well as various drug metabolic processes were inhibited in C2. ATP-binding cassette (ABC) transport proteins play a critical role in drug absorption, distribution, and metabolism, and thus these transport proteins are of considerable pharmacological significance. The pathways by which ESRD affects non-renal drug clearance have been reported to include cytochrome P450 metabolizing enzymes and P-glycoproteins, organic anion-transporting polypeptides, and multidrug resistance-associated protein transport proteins in the liver and gastrointestinal tract (Nolin, 2008). In addition, hepatic and intestinal efflux transport proteins have also been shown to be associated with the pharmacokinetics and pharmacodynamics of drugs during the treatment of osteoporosis (Trdan Lusin et al., 2012). Thus, it has been proposed that these transporter proteins may be part of a larger system of remote communication (“telemetry and signaling”) between cells, organs, body fluid compartments, and possibly even individual organisms. This broader view may help elucidate the link with diabetes, chronic kidney disease, and metabolic syndrome (Nigam, 2015). Our work reinforces this link, and further understanding of the endogenous function of transporter proteins and their involvement in systemic physiology is critical.

### Potential therapeutic target

Currently, bisphosphonates are FDA-approved drugs for the treatment of osteoporosis with antiresorptive properties and are particularly beneficial in individuals with high bone turnover disease, as they both reduce bone turnover. Subgroup analyses of phase three trials have shown therapeutic benefit of bisphosphonates in patients with CKD 1–3b and in patients who have undergone renal transplantation (Jamal et al., 2007; Wilson et al., 2017). However, data in CKD 4-5D are very limited. In addition, anabolic agents may be effective in patients with low bone turnover. Specifically, teriparatide stimulates bone conversion and is approved for the treatment of osteoporosis in the general population (Krege and Wan, 2012), but studies in patients with advanced CDK or ESRD are limited. Our study identified 8 shared genes and 8 shared miRNAs and constructed a common regulatory network based on this for 7 mRNAs and 12 miRNAs in ESRD and OS patients. Lactolipid globule-epidermal growth factor-factor 8 (MFG-E8), also known as lactadherin, is a multifunctional secreted glycoprotein that exhibits multiple functions in cellular physiology affecting health and disease. It plays a role in the clearance of apoptotic cells, anti-inflammation, wound healing, arterial remodeling, and angiogenesis (Li et al., 2013). MFG-E8 deficiency leads to decreased bone mass and accelerates osteoclast-associated bone loss through increased osteoclast production (Sinningen et al., 2015), whereas teriparatide rescues inflammatory bone loss associated with MFG-E8 deficiency (Michalski et al., 2018). In addition, MFG-E8 was significantly upregulated in the diabetic kidney. Silencing MFG-E8 ameliorates renal histological damage by inhibiting phosphorylation of extracellular signal-regulated kinase 1/2 (ERK1⁄2), Akt and glycogen synthase kinase 3beta (GSK-3β) in db/db kidneys (Zhang et al., 2013). Our work shows that hub genes can be used as indicators of OS sensitivity in ESRD patients and therefore MFG-E8 may be an important target for OS management in ESRD patients. miR-194-3p has been shown to be downregulated in osteoporotic samples (Zhou et al., 2022), and in vascular endothelial cells (Li et al., 2021), and vascular endothelial dysfunction is one of the disease features in patients with end-stage renal disease (Kruger et al., 2006), so delivery of miR-194-3p into ESRD patients via bio-nanomaterials may be an effective therapeutic tool for ESRD patients.

### Limitation

Challenges in sample collection have made it difficult to obtain data sets for ESRD with or without OS. We took an alternative approach to characterize the susceptibility of ESRD patients to OS by analyzing samples of CDK development for ESRD and OS samples independently or in combination. In addition, we were unable to experimentally validate the pathogenic role of the identified characteristic key genes and miRNAs in a short period of time, which will be further developed in subsequent studies.

## Conclusion

In conclusion, our study provides key common diagnostic effect genes for ESRD and OS patients, while revealing mechanisms and biological processes that are jointly involved in the disease. In addition, consensus clustering of ESRD based on eight genes successfully distinguished OS-susceptible and non-susceptible populations of ESRD. This study provides new insights to further investigate the molecular mechanisms of ESRD complicated by OS.

## Data Availability

The original contributions presented in the study are included in the article/Supplementary Material, further inquiries can be directed to the corresponding authors.

## References

[B1] Barbie D. A. Tamayo P. Boehm J. S. Kim S. Y. Moody S. E. Dunn I. F. (2009). Systematic RNA interference reveals that oncogenic KRAS-driven cancers require TBK1. Nature 462 (7269), 108–112. 10.1038/nature08460 19847166 PMC2783335

[B2] Broadwell A. Chines A. Ebeling P. R. Franek E. Huang S. Smith S. (2021). Denosumab safety and efficacy among participants in the FREEDOM extension study with mild to moderate chronic kidney disease. J. Clin. Endocrinol. Metab. 106 (2), 397–409. 10.1210/clinem/dgaa851 33211870 PMC7823314

[B3] Bucay N. Sarosi I. Dunstan C. R. Morony S. Tarpley J. Capparelli C. (1998). osteoprotegerin-deficient mice develop early onset osteoporosis and arterial calcification. Genes Dev. 12 (9), 1260–1268. 10.1101/gad.12.9.1260 9573043 PMC316769

[B4] Chen X. Zhi X. Cao L. Weng W. Pan P. Hu H. (2017). Matrine derivate MASM uncovers a novel function for ribosomal protein S5 in osteoclastogenesis and postmenopausal osteoporosis. Cell Death Dis. 8 (9), e3037. 10.1038/cddis.2017.394 28880271 PMC5636967

[B5] Donate-Correa J. Martin-Nunez E. Gonzalez-Luis A. Ferri C. M. Luis-Rodriguez D. Tagua V. G. (2021). Pathophysiological implications of imbalances in fibroblast growth factor 23 in the development of diabetes. J. Clin. Med. 10 (12), 2583. 10.3390/jcm10122583 34208131 PMC8230948

[B6] Evenepoel P. D'Haese P. Brandenburg V. (2015). Sclerostin and DKK1: new players in renal bone and vascular disease. Kidney Int. 88 (2), 235–240. 10.1038/ki.2015.156 26083653

[B7] Ginsberg C. Ix J. H. (2022). Diagnosis and management of osteoporosis in advanced kidney disease: a review. Am. J. Kidney Dis. 79 (3), 427–436. 10.1053/j.ajkd.2021.06.031 34419519

[B8] Hobson S. Arefin S. Kublickiene K. Shiels P. G. Stenvinkel P. (2019). Senescent cells in early vascular ageing and bone disease of chronic kidney disease-A novel target for treatment. Toxins (Basel) 11 (2), 82. 10.3390/toxins11020082 30717151 PMC6409791

[B9] Iimori S. Mori Y. Akita W. Kuyama T. Takada S. Asai T. (2012). Diagnostic usefulness of bone mineral density and biochemical markers of bone turnover in predicting fracture in CKD stage 5D patients--a single-center cohort study. Nephrol. Dial. Transpl. 27 (1), 345–351. 10.1093/ndt/gfr317 21652550

[B10] Isakova T. Nickolas T. L. Denburg M. Yarlagadda S. Weiner D. E. Gutierrez O. M. (2017). KDOQI US commentary on the 2017 KDIGO clinical practice guideline update for the diagnosis, evaluation, prevention, and treatment of chronic kidney disease-mineral and bone disorder (CKD-MBD). Am. J. Kidney Dis. 70 (6), 737–751. 10.1053/j.ajkd.2017.07.019 28941764

[B11] Jamal S. A. Bauer D. C. Ensrud K. E. Cauley J. A. Hochberg M. Ishani A. (2007). Alendronate treatment in women with normal to severely impaired renal function: an analysis of the fracture intervention trial. J. Bone Min. Res. 22 (4), 503–508. 10.1359/jbmr.070112 17243862

[B12] Klawansky S. Komaroff E. Cavanaugh P. F. Jr. Mitchell D. Y. Gordon M. J. Connelly J. E. (2003). Relationship between age, renal function and bone mineral density in the US population. Osteoporos. Int. 14 (7), 570–576. 10.1007/s00198-003-1435-y 12844211

[B13] Kooman J. P. Kotanko P. Schols A. M. Shiels P. G. Stenvinkel P. (2014). Chronic kidney disease and premature ageing. Nat. Rev. Nephrol. 10 (12), 732–742. 10.1038/nrneph.2014.185 25287433

[B14] Krege J. H. Wan X. (2012). Teriparatide and the risk of nonvertebral fractures in women with postmenopausal osteoporosis. Bone 50 (1), 161–164. 10.1016/j.bone.2011.10.018 22036910

[B15] Kruger A. Stewart J. Sahityani R. O'Riordan E. Thompson C. Adler S. (2006). Laser Doppler flowmetry detection of endothelial dysfunction in end-stage renal disease patients: correlation with cardiovascular risk. Kidney Int. 70 (1), 157–164. 10.1038/sj.ki.5001511 16710351

[B16] Langfelder P. Horvath S. (2008). WGCNA: an R package for weighted correlation network analysis. BMC Bioinforma. 9, 559. 10.1186/1471-2105-9-559 PMC2631488 19114008

[B17] Li B. Z. Zhang H. Y. Pan H. F. Ye D. Q. (2013). Identification of MFG-E8 as a novel therapeutic target for diseases. Expert Opin. Ther. Targets 17 (11), 1275–1285. 10.1517/14728222.2013.829455 23972256

[B18] Li Y. Geng Y. Zhou B. Wu X. Zhang O. Guan X. (2021). Long non-coding RNA GAS5 worsens coronary atherosclerosis through MicroRNA-194-3p/TXNIP Axis. Mol. Neurobiol. 58 (7), 3198–3207. 10.1007/s12035-021-02332-x 33638792 PMC8257541

[B19] Liberzon A. Birger C. Thorvaldsdottir H. Ghandi M. Mesirov J. P. Tamayo P. (2015). The Molecular Signatures Database (MSigDB) hallmark gene set collection. Cell Syst. 1 (6), 417–425. 10.1016/j.cels.2015.12.004 26771021 PMC4707969

[B20] Liu T. Zhuang X. X. Qin X. J. Wei L. B. Gao J. R. (2022). Identifying effective diagnostic biomarkers and immune infiltration features in chronic kidney disease by bioinformatics and validation. Front. Pharmacol. 13, 1069810. 10.3389/fphar.2022.1069810 36642989 PMC9838551

[B21] McGeary S. E. Lin K. S. Shi C. Y. Pham T. M. Bisaria N. Kelley G. M. (2019). The biochemical basis of microRNA targeting efficacy. Science 366 (6472), eaav1741. 10.1126/science.aav1741 31806698 PMC7051167

[B22] Michalski M. N. Seydel A. L. Siismets E. M. Zweifler L. E. Koh A. J. Sinder B. P. (2018). Inflammatory bone loss associated with MFG-E8 deficiency is rescued by teriparatide. FASEB J. 32 (7), 3730–3741. 10.1096/fj.201701238R 29475373 PMC5998979

[B23] Newman A. M. Steen C. B. Liu C. L. Gentles A. J. Chaudhuri A. A. Scherer F. (2019). Determining cell type abundance and expression from bulk tissues with digital cytometry. Nat. Biotechnol. 37 (7), 773–782. 10.1038/s41587-019-0114-2 31061481 PMC6610714

[B24] Nigam S. K. (2015). What do drug transporters really do? Nat. Rev. Drug Discov. 14 (1), 29–44. 10.1038/nrd4461 25475361 PMC4750486

[B25] Nolin T. D. (2008). Altered nonrenal drug clearance in ESRD. Curr. Opin. Nephrol. Hypertens. 17 (6), 555–559. 10.1097/MNH.0b013e3283136732 18941346

[B26] Peng H. Yu Y. Gu H. Qi B. Yu A. (2022). MicroRNA-483-5p inhibits osteogenic differentiation of human bone marrow mesenchymal stem cells by targeting the RPL31-mediated RAS/MEK/ERK signaling pathway. Cell Signal 93, 110298. 10.1016/j.cellsig.2022.110298 35248705

[B27] Persy V. Haese P. (2009). Vascular calcification and bone disease: the calcification paradox. Trends Mol. Med. 15 (9), 405–416. 10.1016/j.molmed.2009.07.001 19733120

[B28] Qu X. Huang X. Jin F. Wang H. Hao Y. Tang T. (2013). Bone mineral density and all-cause, cardiovascular and stroke mortality: a meta-analysis of prospective cohort studies. Int. J. Cardiol. 166 (2), 385–393. 10.1016/j.ijcard.2011.10.114 22112679

[B29] Shanahan C. M. (2013). Mechanisms of vascular calcification in CKD-evidence for premature ageing? Nat. Rev. Nephrol. 9 (11), 661–670. 10.1038/nrneph.2013.176 24018414

[B30] Shannon P. Markiel A. Ozier O. Baliga N. S. Wang J. T. Ramage D. (2003). Cytoscape: a software environment for integrated models of biomolecular interaction networks. Genome Res. 13 (11), 2498–2504. 10.1101/gr.1239303 14597658 PMC403769

[B31] Shao J. S. Cheng S. L. Pingsterhaus J. M. Charlton-Kachigian N. Loewy A. P. Towler D. A. (2005). Msx2 promotes cardiovascular calcification by activating paracrine Wnt signals. J. Clin. Invest. 115 (5), 1210–1220. 10.1172/JCI24140 15841209 PMC1077175

[B32] Sharma R. Wu X. Rhodes S. D. Chen S. He Y. Yuan J. (2013). Hyperactive Ras/MAPK signaling is critical for tibial nonunion fracture in neurofibromin-deficient mice. Hum. Mol. Genet. 22 (23), 4818–4828. 10.1093/hmg/ddt333 23863460 PMC3820137

[B33] Sinningen K. Albus E. Thiele S. Grossklaus S. Kurth T. Udey M. C. (2015). Loss of milk fat globule-epidermal growth factor 8 (MFG-E8) in mice leads to low bone mass and accelerates ovariectomy-associated bone loss by increasing osteoclastogenesis. Bone 76, 107–114. 10.1016/j.bone.2015.04.003 25868798

[B34] Sun Y. Byon C. H. Yuan K. Chen J. Mao X. Heath J. M. (2012). Smooth muscle cell-specific runx2 deficiency inhibits vascular calcification. Circ. Res. 111 (5), 543–552. 10.1161/CIRCRESAHA.112.267237 22773442 PMC3678289

[B35] Suzuki K. (2019). Chronic inflammation as an immunological abnormality and effectiveness of exercise. Biomolecules 9 (6), 223. 10.3390/biom9060223 31181700 PMC6628010

[B36] Trdan Lusin T. Mrhar A. Stieger B. Kullak-Ublick G. A. Marc J. Ostanek B. (2012). Influence of hepatic and intestinal efflux transporters and their genetic variants on the pharmacokinetics and pharmacodynamics of raloxifene in osteoporosis treatment. Transl. Res. 160 (4), 298–308. 10.1016/j.trsl.2012.03.002 22683417

[B37] Vervloet M. G. Massy Z. A. Brandenburg V. M. Mazzaferro S. Cozzolino M. Urena-Torres P. (2014). Bone: a new endocrine organ at the heart of chronic kidney disease and mineral and bone disorders. Lancet Diabetes Endocrinol. 2 (5), 427–436. 10.1016/S2213-8587(14)70059-2 24795256

[B38] Wilson L. M. Rebholz C. M. Jirru E. Liu M. C. Zhang A. Gayleard J. (2017). Benefits and harms of osteoporosis medications in patients with chronic kidney disease: a systematic review and meta-analysis. Ann. Intern Med. 166 (9), 649–658. 10.7326/M16-2752 28395318

[B39] Wu P. H. Lin M. Y. Huang T. H. Lee T. C. Lin S. Y. Chen C. H. (2022). Kidney function change and all-cause mortality in denosumab users with and without chronic kidney disease. J. Pers. Med. 12 (2), 185. 10.3390/jpm12020185 35207673 PMC8875658

[B40] Xiao L. Zhong M. Huang Y. Zhu J. Tang W. Li D. (2020). Puerarin alleviates osteoporosis in the ovariectomy-induced mice by suppressing osteoclastogenesis via inhibition of TRAF6/ROS-dependent MAPK/NF-κB signaling pathways. Aging (Albany NY) 12 (21), 21706–21729. 10.18632/aging.103976 33176281 PMC7695364

[B41] Xin Z. Jin C. Chao L. Zheng Z. Liehu C. Panpan P. (2018). A matrine derivative M54 suppresses osteoclastogenesis and prevents ovariectomy-induced bone loss by targeting ribosomal protein S5. Front. Pharmacol. 9, 22. 10.3389/fphar.2018.00022 29441015 PMC5797611

[B42] Yang J. He Q. Wang Y. Pan Z. Zhang G. Liang J. (2022). Gegen Qinlian Decoction ameliorates type 2 diabetes osteoporosis via IGFBP3/MAPK/NFATc1 signaling pathway based on cytokine antibody array. Phytomedicine 94, 153810. 10.1016/j.phymed.2021.153810 34798519

[B43] Zhang B. Wu Q. Li B. Wang D. Wang L. Zhou Y. L. (2020). m6A regulator-mediated methylation modification patterns and tumor microenvironment infiltration characterization in gastric cancer. Mol. Cancer 19 (1), 53. 10.1186/s12943-020-01170-0 32164750 PMC7066851

[B44] Zhang Z. Li B. Y. Li X. L. Cheng M. Yu F. Lu W. D. (2013). Proteomic analysis of kidney and protective effects of grape seed procyanidin B2 in db/db mice indicate MFG-E8 as a key molecule in the development of diabetic nephropathy. Biochim. Biophys. Acta 1832 (6), 805–816. 10.1016/j.bbadis.2013.02.022 23474305

[B45] Zhou H. Jiang J. Chen X. Zhang Z. (2022). Differentially expressed genes and miRNAs in female osteoporosis patients. Med. Baltim. 101 (28), e29856. 10.1097/MD.0000000000029856 PMC11132388 35839011

